# Crystal structure of 3a,6,6,9a-tetra­methyl­dodeca­hydro­naphtho­[2,1-*b*]furan-2-ol

**DOI:** 10.1107/S2056989015016370

**Published:** 2015-09-12

**Authors:** Xin-Wei Shi, Sheng-Kun Li, Dang-Dang Li, Qiang-Qiang Lu

**Affiliations:** a Xi’an Botanical Garden, Institute of Botany of Shaanxi Province, Xi’an 710061, People’s Republic of China; b Lab for Pesticide Synthesis, Department of Pesticide Science, College of Plant Protection, Nanjing Agricultural University, Weigang 1, Xuanwu District, Nanjing 210095, People’s Republic of China

**Keywords:** crystal structure, sclareolide, sclaral, hydrogen bonding

## Abstract

The title compound (common name: sclaral), C_16_H_28_O_2_, is a sclareolide derivative, which was synthesized from sclareolide itself. In the mol­ecule, the two six-membered rings, *A* and *B*, of the labdane skeleton adopt chair conformations and the five-membered O-containing heterocyclic ring *C* displays an envelope conformation, with the methine C atom of the fused C—C bond as the flap. In the crystal, mol­ecules are linked by O—H⋯O hydrogen bonds, forming chains propagating along [100].

## Related literature   

For the chemistry and biological importance of sclareolides and the title compound, see: Dixon *et al.* (2012 ▸); Michaudel *et al.* (2015 ▸); Sun *et al.* (2013 ▸). For previously reported spectroscopic and anal­yt­ical data for the title compound, see: Margaros *et al.* (2007 ▸). For related structures, see: Martínez-Carrera *et al.* (1978 ▸); Huang *et al.* (2008 ▸).
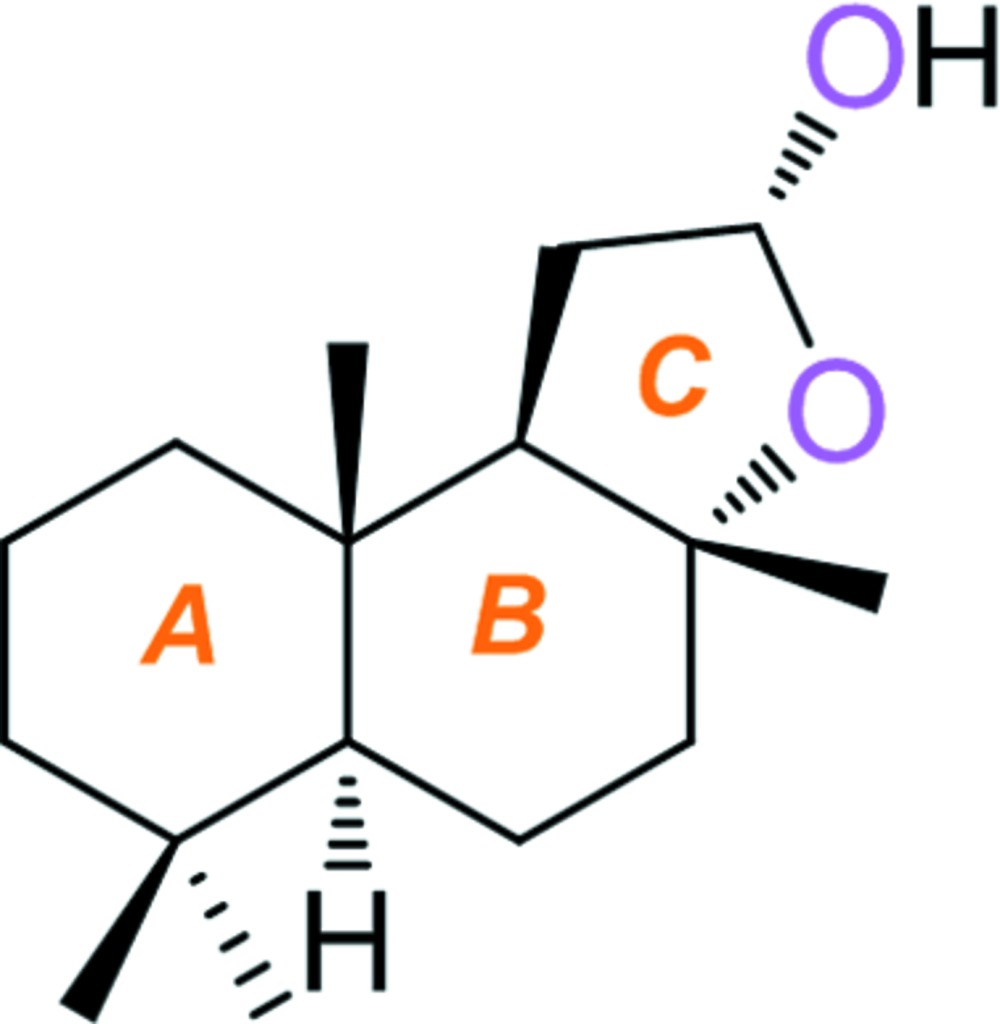


## Experimental   

### Crystal data  

C_16_H_28_O_2_
*M* *_r_* = 252.38
Orthorhombic, 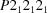

*a* = 7.1675 (8) Å
*b* = 11.2654 (13) Å
*c* = 18.144 (2) Å
*V* = 1465.0 (3) Å^3^
*Z* = 4
Mo *K*α radiation
μ = 0.07 mm^−1^
*T* = 296 K
0.22 × 0.20 × 0.18 mm

### Data collection  

Bruker SMART CCD diffractometer
Absorption correction: multi-scan (*SADABS*; Bruker, 2002 ▸) *T*_min_ = 0.984, *T*_max_ = 0.987
9607 measured reflections
2658 independent reflections
2299 reflections with *I* > 2σ(*I*)
*R*_int_ = 0.030

### Refinement  

*R*[*F* ^2^ > 2σ(*F* ^2^)] = 0.046
*wR*(*F* ^2^) = 0.127
*S* = 1.08
2658 reflections
181 parameters
1 restraint
H atoms treated by a mixture of independent and constrained refinement
Δρ_max_ = 0.40 e Å^−3^
Δρ_min_ = −0.20 e Å^−3^

### 

Data collection: *SMART* (Bruker, 2002 ▸); cell refinement: *SAINT* (Bruker, 2002 ▸); data reduction: *SAINT*; program(s) used to solve structure: *SHELXS97* (Sheldrick, 2008 ▸); program(s) used to refine structure: *SHELXL97* (Sheldrick, 2008 ▸); molecular graphics: *SHELXTL* (Sheldrick, 2008 ▸) and *PLATON* (Spek, 2009 ▸); software used to prepare material for publication: *SHELXTL*.

## Supplementary Material

Crystal structure: contains datablock(s) I, New_Global_Publ_Block. DOI: 10.1107/S2056989015016370/su5201sup1.cif

Structure factors: contains datablock(s) I. DOI: 10.1107/S2056989015016370/su5201Isup2.hkl

Supporting information file. DOI: 10.1107/S2056989015016370/su5201Isup3.cdx

Supporting information file. DOI: 10.1107/S2056989015016370/su5201Isup4.cml

**Fig. 1.**
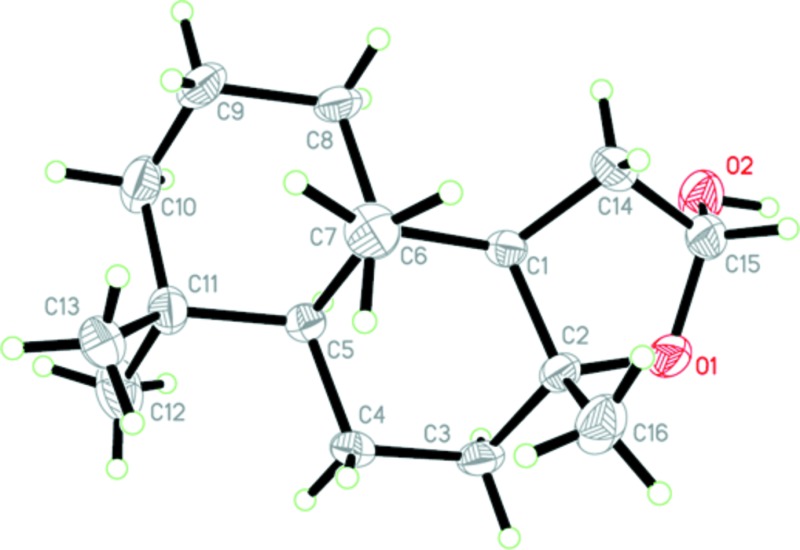
A view of the molecular structure of the title compound, with atom labelling. Displacement ellipsoids are drawn at the 30% probability level.

**Fig. 2.**
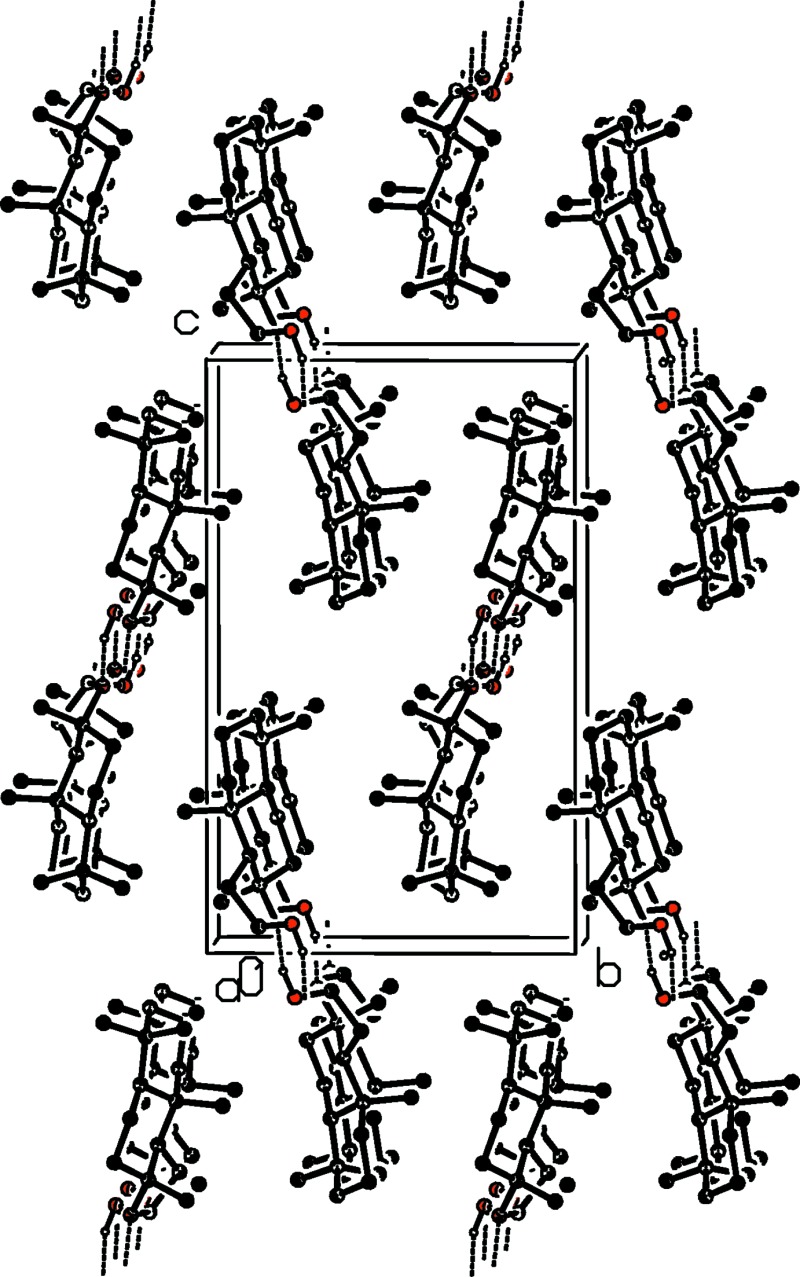
View along the a axis of the crystal packing of the title compound. The hydrogen bonds are shown as dashed lines (see Table 1), and C-bound H atoms have been omitted for clarity.

. DOI: 10.1107/S2056989015016370/su5201fig1.tif
A view of the mol­ecular structure of the title compound, with atom labelling. Displacement ellipsoids are drawn at the 30% probability level.

a . DOI: 10.1107/S2056989015016370/su5201fig2.tif
View along the *a* axis of the crystal packing of the title compound. The hydrogen bonds are shown as dashed lines (see Table 1), and C-bound H atoms have been omitted for clarity.

CCDC reference: 1421899

Additional supporting information:  crystallographic information; 3D view; checkCIF report

## Figures and Tables

**Table 1:** Hydrogen-bond geometry (, )

*D*H*A*	*D*H	H*A*	*D* *A*	*D*H*A*
O2H1*O*O1^i^	0.93(2)	1.90(2)	2.773(2)	155(3)

Symmetry code: (i) 
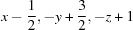
.

## supplementary crystallographic information

### S1. Comment

The title compound, sclaral, is an important reaction inter­mediate in the synthesis of some natural products. It is used as a precursor of borono-sclareolide which makes the direct coupling of a terpenoid "donor" with a non-terpenoid "acceptor" possible (Dixon et al., 2012). It is also used to produce analogs of hongoquercin A, an anti­biotic of fungal origin (Michaudel et al., 2015; Sun et al., 2013). Moreover, sclaral is an inter­mediate in the production of important natural products, such as (+)-premnalane A (Margaros et al., 2007). The title compound has been synthesized and we report herein on its crystal structure,

The molecular structure of the title compound is shown in Fig. 1. The molecule possesses a highly rigid structure, composed of three main rings (A/i>, B and C). The six-membered rings, A/i> (C5/C6/C8—C11) and B (C1—C6), adopt chair conformations, while the five-membered O-containing heterocyclic ring C (C1/C2/C14/C15/O1) displays an envelope conformation, in which atom C1 is the flap.

In the crystal, molecules are linked via O—H···O hydrogen bonds forming chains propagating along the a axis direction (Table 1 and Fig. 2).

### S2. Synthesis and crystallization

A solution of (+)-sclareolide (10.0 g, 40.0 mmol,1.0 eq) in CH_2_Cl_2_ (100ml) was cooled to 195 K and DIBAL-H (1.5M in toluene,32ml, 48.0 mmol, 1.2 eq) was added drop wise over 20 min, and stirring was continued for an additional 60 min. Water was then slowly added until the bubbles vanished then the temperature of the mixture was allow to rise to rt. the mixture was stirred at rt for 30min, and then extracted with CH_2_Cl_2_ (3 × 100 ml). The combined organic extracts were washed with saturated aqueous NaHCO_3_ solution (3 × 50 ml) and washed with brine (3 × 50 ml), dried over MgSO_4_, filtered and concentrated under reduced pressure, affording the title compound, sclaral (yield: 9.37 g, 93 %, 3.7:1 lactol:aldehyde) as a white solid. Spectroscopic and analytical data matches reported previously (Margaros *et al.*, 2007). The white solid was recrystallized from EtOH to afford colourless crystals.

### S3. Refinement

The CH H atoms (H1, H5 and H15) and the hydroxyl H atom (H1O) were located in a difference Fourier map. The CH H atoms were freely refined while the OH H atom was refined with U_iso_(H) = 1.5U_eq_(O). The remaining C-bound H atoms were placed in calculated positions and refined as riding: C—H = 0.96-0.97 Å with U_iso_(H) = 1.5U_eq_(C) for methyl H atoms and 1.2U_eq_(C) for other H atoms.

### Crystal data

**Table:**

C_16_H_28_O_2_	*F*(000) = 560
*M**_r_* = 252.38	*D*_x_ = 1.144 Mg m^−^^3^
Orthorhombic, *P*2_1_2_1_2_1_	Mo *K*α radiation, λ = 0.71073 Å
Hall symbol: P 2ac 2ab	Cell parameters from 2540 reflections
*a* = 7.1675 (8) Å	θ = 3.1–21.6°
*b* = 11.2654 (13) Å	µ = 0.07 mm^−^^1^
*c* = 18.144 (2) Å	*T* = 296 K
*V* = 1465.0 (3) Å^3^	Block, colourless
*Z* = 4	0.22 × 0.20 × 0.18 mm

### Data collection

**Table:**

Bruker SMART CCD diffractometer	2658 independent reflections
Radiation source: fine-focus sealed tube	2299 reflections with *I* > 2σ(*I*)
Graphite monochromator	*R*_int_ = 0.030
phi and ω scans	θ_max_ = 25.3°, θ_min_ = 2.1°
Absorption correction: multi-scan (*SADABS*; Bruker, 2002)	*h* = −8→8
*T*_min_ = 0.984, *T*_max_ = 0.987	*k* = −13→13
9607 measured reflections	*l* = −21→16

### Refinement

**Table:**

Refinement on *F*^2^	Primary atom site location: structure-invariant direct methods
Least-squares matrix: full	Secondary atom site location: difference Fourier map
*R*[*F*^2^ > 2σ(*F*^2^)] = 0.046	Hydrogen site location: inferred from neighbouring sites
*wR*(*F*^2^) = 0.127	H atoms treated by a mixture of independent and constrained refinement
*S* = 1.08	*w* = 1/[σ^2^(*F*_o_^2^) + (0.0651*P*)^2^ + 0.258*P*] where *P* = (*F*_o_^2^ + 2*F*_c_^2^)/3
2658 reflections	(Δ/σ)_max_ < 0.001
181 parameters	Δρ_max_ = 0.40 e Å^−^^3^
1 restraint	Δρ_min_ = −0.19 e Å^−^^3^

### Special details

**Table:**

Geometry. All e.s.d.'s (except the e.s.d. in the dihedral angle between two l.s. planes) are estimated using the full covariance matrix. The cell e.s.d.'s are taken into account individually in the estimation of e.s.d.'s in distances, angles and torsion angles; correlations between e.s.d.'s in cell parameters are only used when they are defined by crystal symmetry. An approximate (isotropic) treatment of cell e.s.d.'s is used for estimating e.s.d.'s involving l.s. planes.
Refinement. Refinement of *F*^2^ against ALL reflections. The weighted *R*-factor *wR* and goodness of fit *S* are based on *F*^2^, conventional *R*-factors *R* are based on *F*, with *F* set to zero for negative *F*^2^. The threshold expression of *F*^2^ > σ(*F*^2^) is used only for calculating *R*-factors(gt) *etc*. and is not relevant to the choice of reflections for refinement. *R*-factors based on *F*^2^ are statistically about twice as large as those based on *F*, and *R*- factors based on ALL data will be even larger.

### Fractional atomic coordinates and isotropic or equivalent isotropic displacement parameters (Å^2^)

**Table:**

	*x*	*y*	*z*	*U*_iso_*/*U*_eq_	
C1	0.0482 (3)	0.86847 (19)	0.67801 (12)	0.0348 (5)	
C2	0.2058 (3)	0.8474 (2)	0.62309 (12)	0.0392 (5)	
C3	0.3421 (3)	0.7594 (2)	0.65591 (13)	0.0469 (6)	
H3A	0.4488	0.7504	0.6235	0.056*	
H3B	0.2825	0.6826	0.6612	0.056*	
C4	0.4067 (3)	0.8050 (2)	0.73202 (13)	0.0443 (5)	
H4A	0.4901	0.7471	0.7539	0.053*	
H4B	0.4759	0.8783	0.7255	0.053*	
C5	0.2429 (3)	0.82737 (18)	0.78462 (12)	0.0342 (5)	
C6	0.1029 (3)	0.91962 (17)	0.75332 (11)	0.0339 (5)	
C7	0.1778 (4)	1.04780 (19)	0.74731 (14)	0.0515 (6)	
H7A	0.1100	1.0896	0.7098	0.077*	
H7B	0.3078	1.0459	0.7347	0.077*	
H7C	0.1620	1.0875	0.7937	0.077*	
C8	−0.0711 (3)	0.9208 (3)	0.80299 (14)	0.0519 (6)	
H8A	−0.1541	0.9836	0.7869	0.062*	
H8B	−0.1368	0.8460	0.7976	0.062*	
C9	−0.0233 (4)	0.9397 (3)	0.88443 (15)	0.0655 (8)	
H9A	0.0283	1.0186	0.8910	0.079*	
H9B	−0.1363	0.9343	0.9136	0.079*	
C10	0.1147 (4)	0.8493 (3)	0.91114 (14)	0.0620 (8)	
H10A	0.0560	0.7717	0.9097	0.074*	
H10B	0.1444	0.8665	0.9622	0.074*	
C11	0.2970 (3)	0.8434 (2)	0.86729 (13)	0.0462 (6)	
C12	0.4061 (5)	0.7330 (3)	0.89311 (17)	0.0775 (9)	
H12A	0.5323	0.7377	0.8756	0.116*	
H12B	0.3480	0.6628	0.8738	0.116*	
H12C	0.4059	0.7298	0.9460	0.116*	
C13	0.4176 (4)	0.9519 (3)	0.88354 (15)	0.0579 (7)	
H13A	0.5198	0.9547	0.8494	0.087*	
H13B	0.4652	0.9468	0.9329	0.087*	
H13C	0.3438	1.0226	0.8786	0.087*	
C14	−0.1012 (4)	0.9213 (2)	0.62815 (14)	0.0520 (6)	
H14A	−0.0739	1.0033	0.6157	0.062*	
H14B	−0.2240	0.9166	0.6504	0.062*	
C15	−0.0850 (3)	0.8402 (2)	0.56099 (14)	0.0495 (6)	
H15	−0.112 (4)	0.884 (2)	0.5155 (15)	0.059*	
C16	0.3106 (4)	0.9544 (3)	0.59153 (15)	0.0622 (7)	
H16A	0.3743	0.9313	0.5472	0.093*	
H16B	0.3997	0.9823	0.6270	0.093*	
H16C	0.2234	1.0166	0.5805	0.093*	
H1	0.007 (3)	0.7879 (18)	0.6884 (10)	0.024 (5)*	
H5	0.168 (3)	0.751 (2)	0.7848 (12)	0.040 (6)*	
O1	0.1024 (2)	0.79612 (16)	0.56104 (8)	0.0520 (5)	
O2	−0.2051 (3)	0.7454 (2)	0.56902 (11)	0.0678 (6)	
H1O	−0.242 (4)	0.715 (3)	0.5234 (11)	0.081*	

### Atomic displacement parameters (Å^2^)

**Table:**

	*U*^11^	*U*^22^	*U*^33^	*U*^12^	*U*^13^	*U*^23^
C1	0.0291 (10)	0.0358 (11)	0.0396 (11)	0.0007 (9)	0.0009 (9)	−0.0037 (9)
C2	0.0342 (10)	0.0491 (12)	0.0342 (11)	−0.0040 (10)	0.0055 (10)	−0.0055 (10)
C3	0.0352 (11)	0.0571 (14)	0.0485 (14)	0.0072 (11)	0.0082 (11)	−0.0129 (11)
C4	0.0313 (10)	0.0529 (13)	0.0485 (14)	0.0097 (10)	0.0001 (10)	−0.0055 (10)
C5	0.0331 (10)	0.0328 (10)	0.0368 (11)	−0.0020 (9)	0.0018 (9)	−0.0018 (9)
C6	0.0270 (9)	0.0355 (10)	0.0393 (11)	0.0012 (9)	0.0011 (9)	−0.0073 (9)
C7	0.0607 (15)	0.0336 (12)	0.0601 (15)	0.0041 (11)	−0.0055 (13)	−0.0088 (11)
C8	0.0304 (11)	0.0749 (16)	0.0503 (14)	0.0045 (11)	0.0059 (11)	−0.0221 (13)
C9	0.0430 (13)	0.104 (2)	0.0498 (15)	−0.0091 (15)	0.0137 (12)	−0.0322 (16)
C10	0.0613 (16)	0.090 (2)	0.0352 (13)	−0.0243 (16)	0.0041 (13)	−0.0063 (13)
C11	0.0474 (13)	0.0528 (13)	0.0385 (12)	−0.0035 (11)	−0.0058 (11)	−0.0025 (10)
C12	0.092 (2)	0.078 (2)	0.0620 (18)	0.0127 (19)	−0.0247 (18)	0.0158 (15)
C13	0.0434 (13)	0.0751 (17)	0.0551 (15)	−0.0065 (13)	−0.0103 (13)	−0.0149 (14)
C14	0.0439 (13)	0.0601 (15)	0.0520 (14)	0.0096 (12)	−0.0092 (12)	−0.0063 (12)
C15	0.0407 (12)	0.0673 (15)	0.0404 (13)	−0.0035 (12)	−0.0050 (11)	−0.0024 (12)
C16	0.0648 (16)	0.0754 (18)	0.0463 (14)	−0.0191 (15)	0.0090 (14)	0.0086 (13)
O1	0.0417 (8)	0.0749 (11)	0.0393 (9)	−0.0020 (9)	0.0020 (8)	−0.0166 (8)
O2	0.0591 (11)	0.0950 (15)	0.0494 (11)	−0.0205 (11)	−0.0057 (10)	−0.0045 (10)

### Geometric parameters (Å, º)

**Table:**

C1—C14	1.523 (3)	C6—C8	1.539 (3)
C1—C2	1.525 (3)	C6—C7	1.544 (3)
C1—C6	1.534 (3)	C8—C9	1.532 (4)
C2—O1	1.466 (3)	C9—C10	1.500 (4)
C2—C3	1.513 (3)	C10—C11	1.531 (3)
C2—C16	1.531 (3)	C11—C13	1.526 (3)
C3—C4	1.544 (3)	C11—C12	1.542 (4)
C4—C5	1.534 (3)	C14—C15	1.527 (3)
C5—C6	1.552 (3)	C15—O2	1.380 (3)
C5—C11	1.560 (3)	C15—O1	1.432 (3)
			
C14—C1—C2	101.16 (18)	C1—C6—C5	103.87 (16)
C14—C1—C6	124.19 (19)	C8—C6—C5	108.38 (18)
C2—C1—C6	116.81 (16)	C7—C6—C5	115.30 (17)
O1—C2—C3	111.75 (18)	C9—C8—C6	112.63 (18)
O1—C2—C1	100.87 (15)	C10—C9—C8	111.4 (2)
C3—C2—C1	108.84 (18)	C9—C10—C11	115.1 (2)
O1—C2—C16	105.71 (18)	C13—C11—C10	110.4 (2)
C3—C2—C16	110.26 (19)	C13—C11—C12	107.4 (2)
C1—C2—C16	119.00 (19)	C10—C11—C12	108.0 (2)
C2—C3—C4	109.12 (18)	C13—C11—C5	114.8 (2)
C5—C4—C3	112.42 (17)	C10—C11—C5	107.00 (18)
C4—C5—C6	112.15 (17)	C12—C11—C5	109.0 (2)
C4—C5—C11	115.27 (17)	C1—C14—C15	100.75 (19)
C6—C5—C11	115.79 (17)	O2—C15—O1	108.5 (2)
C1—C6—C8	108.51 (17)	O2—C15—C14	109.4 (2)
C1—C6—C7	112.17 (19)	O1—C15—C14	106.16 (19)
C8—C6—C7	108.36 (19)	C15—O1—C2	109.75 (17)

### Hydrogen-bond geometry (Å, º)

**Table:**

*D*—H···*A*	*D*—H	H···*A*	*D*···*A*	*D*—H···*A*
O2—H1*O*···O1^i^	0.93 (2)	1.90 (2)	2.773 (2)	155 (3)

Symmetry code: (i) *x*−1/2, −*y*+3/2, −*z*+1.
