# Supplementary figures and images for: Crystal structure of 3a,6,6,9a-tetra­methyl­dodeca­hydro­naphtho­[2,1-b]furan-2-ol
Source: Acta Crystallogr E Crystallogr Commun. 2015 Sep 12;71(Pt 10):o710–1. doi: 10.1107/S2056989015016370 (PMC4647384; doi:10.1107/S2056989015016370)

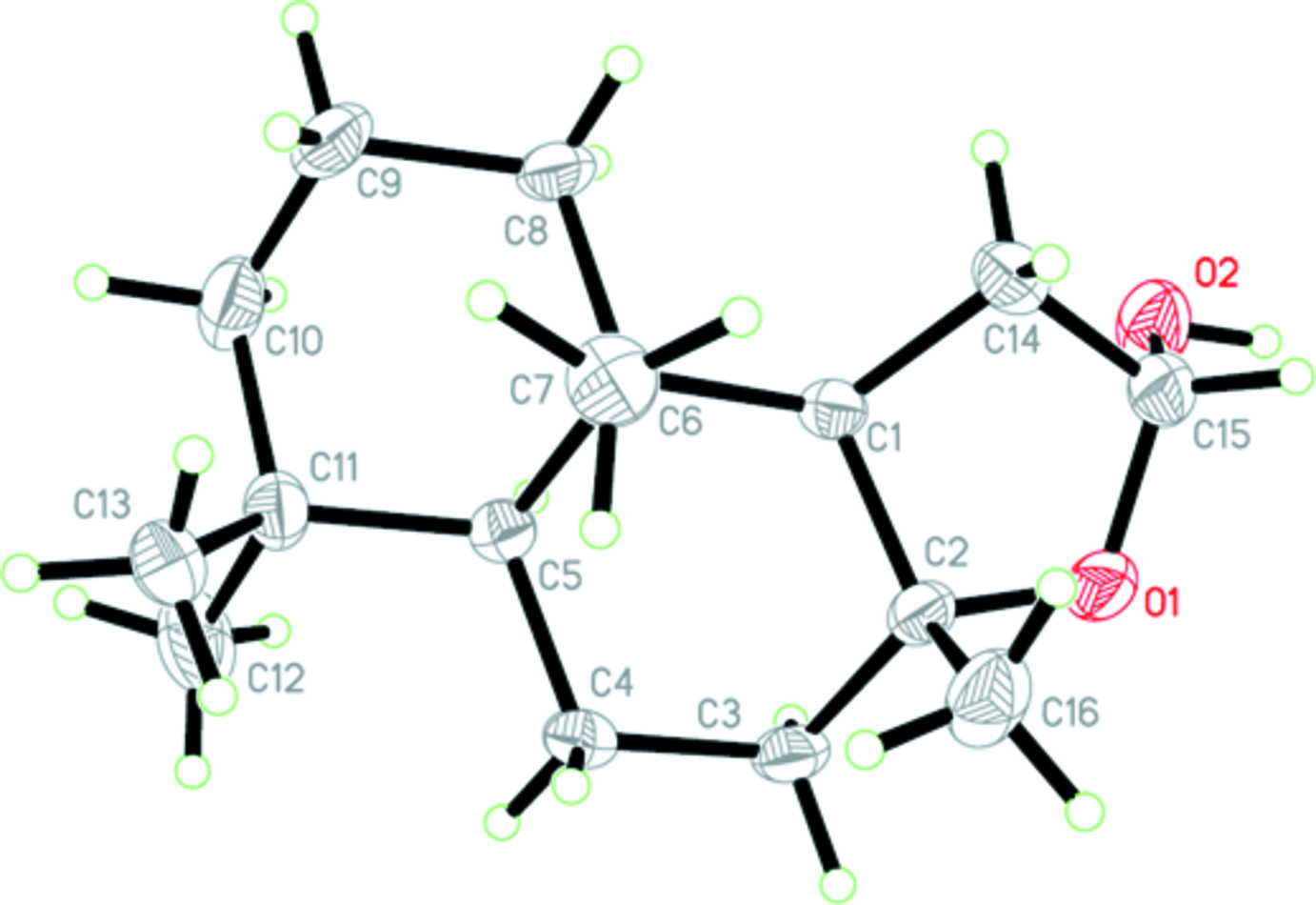

Supplement: Supplementary file 5 [file e-71-0o710-fig1.tif]

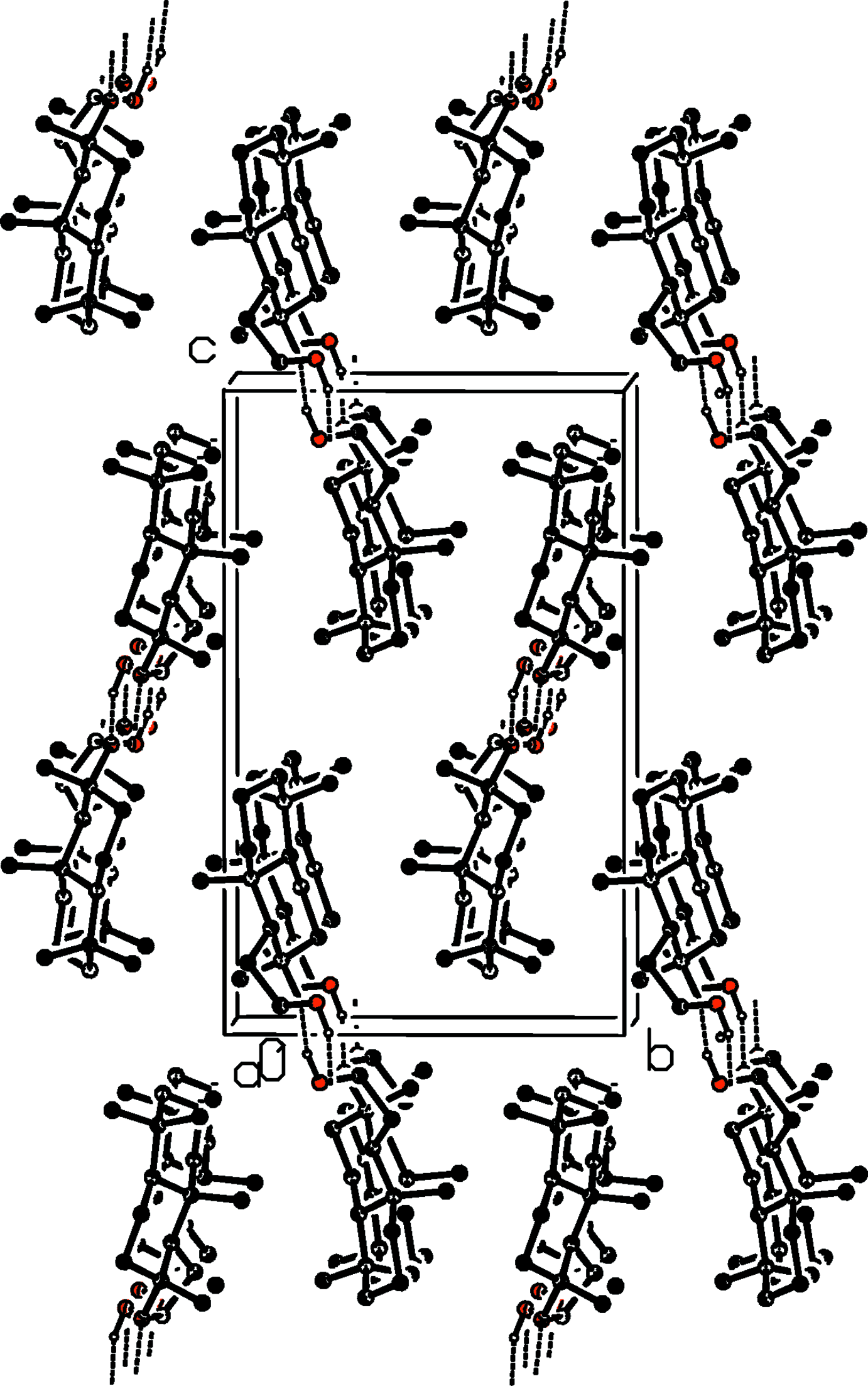

Supplement: Supplementary file 6 [file e-71-0o710-fig2.tif]
